# Application of the Intervention Mapping Framework to Develop an Integrated Twenty-First Century Core Curriculum—Part 1: Mobilizing the Community to Revise the Masters of Public Health Core Competencies

**DOI:** 10.3389/fpubh.2017.00287

**Published:** 2017-11-01

**Authors:** Rita DeBate, Jaime A. Corvin, Kate Wolfe-Quintero, Donna J. Petersen

**Affiliations:** ^1^Department of Health Policy and Management, College of Public Health, University of South Florida, Tampa, FL, United States; ^2^Department of Global Health, College of Public Health, University of South Florida, Tampa, FL, United States; ^3^College of Public Health, University of South Florida, Tampa, FL, United States

**Keywords:** public health, Masters of Public Health foundational core, competencies, experiential learning, pedagogy

## Abstract

Twenty-first century health challenges have significantly altered the expanding role and functions of public health professionals. Guided by a call from the Association of Schools and Programs of Public Health’s (ASPPH) and the *Framing the Future: The Second 100 Years of Education for Public Health* report to adopt new and innovative approaches to prepare public health leaders, the University of South Florida College of Public Health aimed to self-assess the current Masters of Public Health (MPH) core curriculum with regard to preparing students to meet twenty-first century public health challenges. This paper describes how Intervention Mapping was employed as a framework to increase readiness and mobilize the COPH community for curricular change. Intervention Mapping provides an ideal framework, allowing organizations to access capacity, specify goals, and guide the change process from curriculum development to implementation and evaluation of competency-driven programs. The steps outlined in this paper resulted in a final set of revised MPH core competencies that are interdisciplinary in nature and fulfill the emergent needs to address changing trends in both public health education and challenges in population health approaches. Ultimately, the competencies developed through this process were agreed upon by the entire College of Public Health faculty, signaling one college’s readiness for change, while providing the impetus to revolutionize the delivery of public health education at the University of South Florida.

This article is *Part 1* of a series of 3 articles published in Frontiers of Public Health. Other articles include:

Part 2: Application of the Intervention Mapping Framework to Develop an Integrated 21st Century Core Curriculum—Translation of MPH Core Competencies into an Integrated Theory-based Core Curriculum (1).
Part 3: Application of the Intervention Mapping Framework to Develop an Integrated 21st Century Core Curriculum—Curriculum Implementation and Evaluation (2).

## Background and Rationale

Twenty-first century health challenges have significantly altered the expanding role and functions of public health professionals. As the 2002 Institute of Medicine (IOM) report noted, contemporary public health training needs require a twenty-first century approach to public health education (3). In response to this challenge, and corresponding with the 100 year anniversary of the Welch-Rose Report of 1915, the Association of Schools and Programs of Public Health (ASPPH) established an expert public health task force to investigate new public health training demands, and develop guidelines for redesigning public health curricula (4–6). At the cornerstone of public health education, the resulting *Framing the Future: The Second 100 Years of Education for Public Health* report recommends that Masters of Public Health (MPH) training programs incorporate carefully sequenced, applied, skills-based, and interdisciplinary programs of study that are clearly differentiated from the BSPH and DrPH, as well as new content and skills pertaining to systems thinking, public health-specific communication, social marketing, cultural competency, public health biology, globalization, and leadership (7).

In this special issue of *Frontiers in Public Health Education and Promotion*, we have assembled a three-part series of original articles that provide examples and insights pertaining to how the College of Public Health (COPH) at the University of South Florida (USF), an accredited school of public health, transformed a traditional MPH core curriculum into an integrated curriculum based on the content and skill-based knowledge required for training the next generation of twenty-first century public health practitioners. Intervention Mapping was the conceptual framework employed to guide the curricular revisions (8). To that end, each manuscript in the series focuses on the application of this framework and how the associated steps were employed to guide the curricular revision. This paper, the first in the series, presents the call to action and discusses how we mobilized the college community to develop integrated twenty-first century MPH core competencies. Part 2 details the methods used to translate these MPH core competencies into an integrated core curriculum. Lastly, Part 3 describes implementation methods and evaluation design, while also outlining preliminary outcomes. Within each paper, we describe implications for applying the Intervention Mapping framework for curricular revisions and lessons learned.

Revision of a traditional curriculum is always a challenging mixture of community awareness and mobilization, curricular design based on evidence-informed pedagogical practices, planning for sustainability, implementation strategies, and evaluation. Far from a simple process, as evidenced by the articles in this issue, this complex, multistep transformation requires a dedicated team, critical review, and detailed analysis at every stage. Thus, we believe strategies and lessons learned from this endeavor can provide insight into redesigning an MPH program and implementing an updated curriculum designed to better meet changing twenty-first century public health needs. It is our hope that the articles presented in this special series will add to the existing knowledge of pedagogy in public health, while also furthering discussion with regard to the advancement of MPH curriculum development, implementation, and evaluation, as well as the professionalization of the degree program, ultimately resulting in a strong public health workforce.

## The Learning Environment

The COPH at the USF was founded in statute by the Florida Legislature in 1984 as the first school of public health in the State of Florida. The COPH is fully accredited by the Council on Education for Public Health (CEPH) and comprises five academic departments, including Community and Family Health, Environmental and Occupational Health, Epidemiology and Biostatistics, Global Health, and Health Policy and Management. Shadowing the conventional MPH core curricula, the COPH has traditionally offered five independent courses as the core of the MPH program: Epidemiology, Biostatistics, Environmental and Occupational Health, Health Policy and Management, and Social and Behavioral Sciences.

Guided by the ASPPH challenge to reevaluate the way public health is taught and *Framing the Future: The Second 100 Years of Education for Public Health’s* call for adopting innovative approaches to better prepare tomorrow’s public health leaders, the Dean of the USF COPH charged the faculty to self-assess the current MPH core curriculum with regard to preparing students to meet twenty-first century public health challenges (7). To meet this charge, a review committee comprised of administrative leaders, faculty from each department, instructors of current core courses, educational support staff, and students was appointed. This committee, known as the transforming the MPH (TMPH) committee, convened several meetings and adopted Intervention Mapping as the framework to guide the transformation process and to inform the resulting pedagogical framework.

## Intervention Mapping for Curriculum Design

Intervention Mapping is a framework for implementing theory and evidence-informed decision-making in planning, implementing, and evaluating system-based programs (8). While applying public health theory and evidence-based approaches has been widely encouraged in the development of public health curricula, the translation of theoretical constructs and principles pertaining to teaching and learning has not been well described. This paper attempts to fill that gap by presenting how the University of South Florida College of Public Health mobilized to develop integrated twenty-first century MPH core competencies and how Intervention Mapping was used to guide the development of its transformed curriculum.

Intervention mapping is an iterative planning approach typically used to assist with the development of interventions (8). However, this systematic approach can be applied to any program development effort. Intervention Mapping is a useful framework for guiding decision making with regard to identifying which theory(ies) and constructs within larger theoretical frameworks are most effective for various cognitive, skill, and affective learning objectives, while also informing when to use theories to guide decisions with regard to curriculum mapping and specific lesson plans. Intervention Mapping also guides processes for assessing the validity and strength of available evidence, translating competencies to teaching and learning strategies, informing the use of various modes of teaching and learning methods, and apprising the complexity of multifactorial health issues requiring multilevel curricular development (8).

As shown in Table 1, Intervention Mapping is a systematic process comprised of six process-based steps from which the theory- and evidence-based intervention and evaluation plans are developed. These steps require examination and actions related to needs assessment, matrices of change, theories and methods, programs, implementation, and evaluation. When this framework is applied to curriculum development, Step 1 requires an assessment of college and faculty capacity and specification of curricular goals and learning outcomes. Step 2 then focuses on the creation of matrices that specify competencies and performance objectives as well as learning determinants. Step 3 establishes the theories and methods that will be applied to achieve these outcomes. These first three steps provide a foundation for Step 4, which is development of a curricular map that establishes the courses, modules, and lessons to be delivered in the new program. Step 5 specifies how the teaching and learning plans are implemented and sustained by the instructors (i.e., implementers) and the students (i.e., adopters). Lastly, Step 6 develops the process, impact, and outcome evaluation plans. The current article describes how the authors employed Step 1 of the Intervention Mapping framework to increase readiness and mobilize the COPH community for curricular change.

**Table 1:** Intervention Mapping framework and translation to curriculum development.

Step	Intervention Mapping Framework	Translation to Curriculum Development
1	*Conduct a Needs Assessment* Assess system capacity Specify health and quality of life intervention outcomes	*Conduct a Needs Assessment* Assess university, college, and faculty system capacity Specify curricular goals and learning outcomes
2	*Create Matrices of Change Objectives* Specify behavioral and environmental change objectives State performance objectives Select determinants	*Create Matrices of Change Objectives* Specify knowledge, skill, and affective objectives State performance objectives Select learning determinants
3	*Select theory-based methods and practical applications* Generate intervention ideas with the research/planning team Identify theoretical methods Choose intervention methods Select and design practical applications Ensure practical applications address specified change objectives	*Select theory-based teaching methods and practical applications* Generate teaching and learning ideas with the research/planning team Identify theoretical teaching methods Choose teaching/learning methods Select and design practical applications Ensure practical applications address specified learning objectives
4	*Organize methods and applications into an intervention program* Create intervention themes, scope, sequence, and materials Prepare and design documents Review available program materials Draft intervention materials and protocols Pretest intervention materials and protocols Produce materials and protocols	*Organize methods and applications into a curricular map* Create curricular courses, themes, modules, sequence, and materials Prepare and design lesson plans Review available program materials Draft curriculum materials and protocols Pretest curricular materials and protocols Produce curricular materials and protocols
5	*Plan for program adoption, implementation, and sustainability* Identify potential intervention adopters and implementers State intervention use outcomes and performance objectives Specify determinants for adoption and implementation Create a matrix of change objectives Select methods and practical applications Design interventions for adoption and implementation	*Plan for curriculum adoption, implementation, and sustainability* Identify potential students and instructors State potential instructor outcomes and performance objectives Specify determinants for instructor adoption and implementation Create a matrix of change objectives Select methods and practical applications Design interventions for instructor adoption and implementation
6	*Generate an evaluation plan* Review program logic model Develop evaluation questions Write process evaluation questions Develop indicators and measures Specify evaluation design	*Generate an evaluation plan* Review curriculum map Develop evaluation questions Write process evaluation questions Develop indicators and measures Specify evaluation design

## Methods

### Intervention Mapping Step 1a: Assess University, College, and Faculty System Capacity

Mirroring the committee charge, the first step in the Intervention Mapping framework consisted of conducting a needs assessment. The TMPH committee began by assessing the college and university system capacity to support and sustain a revised core curriculum by reviewing all available data. The committee focused on data from the prior 5 years including: (a) student demographics and 5-year trends; (b) core course student evaluations; (c) student exit surveys; and (d) student pass rates on the Certified in Public Health (CPH) exam. The committee also reviewed faculty demographics and readiness indicators, as well as university system policies regarding credit hours, course scheduling, and graduation requirements. The TMPH committee took into consideration the COPH’s guiding mission, vision, core values, and strategic plan, along with numerous national and professional reports, including the ASPPH MPH Core Competency Model version 2.3 (9); the National Board of Public Health Examiners CPH exam content (10); the ASPPH report on employer trends (4); the CEPH accreditation standards on public health education (11); and the ASPPH Framing the Future draft report on the MPH as it became available (5). The initial objective was to “take the temperature” of the current state of the College with regard to twenty-first century public health knowledge, skills, and teaching strategies and assess feasibility, desirability, readiness, and capacity for transforming the traditional MPH core curriculum.

Following a thorough review of the data and with regard to the College’s guiding principles, the TMPH committee identified several key factors that supported changes to current MPH core curriculum including: (a) a gradual increase in number of students and concentrations across the college and corresponding increase in faculty teaching responsibilities; (b) data indicating that students took the traditional core courses in a random order, often delayed until the end of their program rather than as the foundational courses they were intended to be; (c) student feedback that the current core courses offered content that was redundant and included very few experiential learning opportunities; (d) student feedback that the activities of the Capstone course, which required students to integrate and apply public health knowledge in interdisciplinary teams, should come earlier in the program; (e) faculty feedback that students needed more room in the curriculum for advanced practice electives; (e) employer feedback that students did not graduate with enough skills in public health practice, communication, or leadership; and (f) recognition that educational and technological paradigms were changing and needed to be incorporated into any new curricula. In summary, the data confirmed that changes to the existing core were both necessary and feasible, while guiding principles urged delivery as a series of integrated learning experiences rather than as a set of distinct core disciplines.

To enlist feedback from the faculty, the TMPH committee took these ideas back for discussion within the five college departments. The Dean also held a series of “Coffee & Conversations” with faculty to discuss the direction of the college and its programs and ensure cross-disciplinary discussion. Following these discussions, the TMPH committee reviewed all feedback and developed an initial formative assessment proposal which summarized their analysis of both the qualitative and quantitative data and reports, and provided recommendations for curricular goals and guiding principles (e.g., key considerations, design features, and critical content) that would inform transformation of the MPH core. To gage faculty readiness, the proposal was presented to the college faculty and staff through a series of town hall meetings where faculty were encouraged to ask questions, discuss beliefs, debate issues, and provide valuable feedback. After a series of lively discussions, debates, and revisions, a final set of principles was agreed upon. These principles, outlined in Table 2, guided the subsequent competency development process as described below.

**Table 2:** University of South Florida College of Public Health guiding principles for Masters of Public Health (MPH) core curriculum revisions.

Aligns with the following key points from the Association of Schools and Programs of Public Health (ASPPH) Framing the Future report: *Key considerations*: Should be clearly distinguished from the BSPH and the DrPH. Interdisciplinary delivered, rigorous, applied, skill-based, competency based. Attention to public health core values. Strong connections to applied public health practice. Global health perspectives and content should be covered in all MPH degrees. *Design features*: Minimum number of credits should not be increased beyond 42. MPH core should be no more than one-third of the content or credits of the MPH degree. Rigorous, structured, and carefully sequenced curriculum. The core can be delivered as a series of integrated learning experiences rather than as a set of distinct courses in the traditional core disciplines. Concentrations should have depth. Practicum and culminating experience should be considered primarily as elements of the concentration rather than the core. MPH degree should have distinct and defined learning objectives for each of its major elements, including core, concentration, practicum, and culminating experience. *Critical Content*: History and philosophy of public health as well as its core values, concepts, functions, and leadership roles. Concepts, methods, and tools of public health data collection, analysis and interpretation, and the evidence-based reasoning and informatics approaches that are essential to public health practice. Population health concepts, and the processes, approaches, and interventions that identify and address the major health-related needs and concerns of populations. Identification and pursuit of opportunities for promoting health and preventing disease across the life span and for enhancing public health preparedness. Biological, environmental, socioeconomic, behavioral, cultural, and other factors that impact human health influence the global and societal burden of disease and contribute to health disparities. The cultural context of public health issues and respectful engagement with people of different cultures and socioeconomic strata. Principles of effective functioning within and across organizations and as members of interdisciplinary and inter-professional teams. Concepts of project implementation and management, including planning, budgeting, human resources, assessment, and evaluation. Characteristics and organizational structures of the U.S. health care system and how they compare to health care systems in other countries. Legal, ethical, economic, and regulatory dimensions of health care and public health policy, the roles, influences, and responsibilities of the different agencies and branches of government, and approaches to developing, evaluating, and advocating for public health policies. Public health-specific communication and social marketing, including technical and professional writing and the use of mass media and electronic technology. Globalization and sustainable development and their relationship to population health. Aligns with the National Board of Public Health Examiners, Certified in Public Health (CPH) Exam Content Outline: General principles Biostatistics Health Policy and Management Environmental Health Sciences Epidemiology Social and Behavioral Sciences Communication and Informatics Diversity and Culture Leadership Ethics and Professionalism Program Planning and Evaluation Public Health Biology Systems Thinking Draws from the ASPH Master’s Degree in Public Health Core Competency Model Version 2.3. Aligns with COPH mission, vision, core values. Aligns with COPH and USF systems. Addresses student demographics and student needs. Draws from Experiential Learning curriculum design principles, information processing theories, and learning theories.

### Intervention Mapping Step 1b: Specify Curricular Goals and Learning Outcomes

Through iterative conversations with the COPH faculty, general consensus emerged regarding the need for an interdisciplinary, experiential, and crosscutting core MPH curriculum that students could complete as a cohort at the beginning of their program. To explore this further, the TMPH committee tasked a subcommittee of interdisciplinary faculty with mapping the MPH competencies to determine what interdisciplinary integration would look like. To complete this task, the TMPH Curriculum Development Committee utilized both the college MPH core competencies and the ASPPH version 2.3 core and crosscutting competencies (9) to create competency concept maps. Utilizing a modified pile-sorting method, competencies were sorted, themes began to emerge, and redundancies were identified. The process continued until all redundancies were eliminated and cohesive themes were identified and agreed upon. The subsequent concept maps were used to draft interdisciplinary core competencies for a transformed MPH.

To encourage faculty readiness and support for these potential changes, a modified mini-Delphi method was used in face-to-face meetings with faculty. During the meetings, faculty reviewed the competencies and commented on wording, content, and level of Blooms Taxonomy each competency was intended to address. A member of the TMPH curriculum development committee acted as the facilitator by collecting and analyzing feedback to identify common and conflicting viewpoints regarding each proposed competency. The process of reviewing and analyzing covered several rounds until synthesis and consensus was met. The final set of revised MPH core competencies was presented to the faculty for review and comment prior to moving to the next step of the Intervention Mapping process. Table 3 presents the resulting MPH core competencies. These competencies drove the transformation of the MPH curriculum at the USF and, we believe, are better reflective of the needs of the twenty-first century public health student and practitioner.

**Table 3:** University of South Florida College of Public Health final integrated Masters of Public Health core competencies.

Examine the history and philosophy of public health as well as its core values, concepts, functions, and leadership roles. Compare and contrast characteristics and organizational structures of the U.S. health system to health care systems in other countries. Describe legal, ethical, economic, and regulatory dimensions of health care and public health policy; the roles, influences, and responsibilities of the different agencies and branches of government; and approaches to developing, evaluating, and advocating for public health policies. Recognize biological, environmental, socioeconomic, behavioral, cultural, and other factors that impact human health, influence the global and societal burden of disease, and contribute to health disparities across the lifespan. Describe globalization and sustainable development and their relationship to population health. Examine population health concepts, and the processes, approaches, and interventions that identify and address the major health-related needs and concerns of populations across the lifespan and for enhancing public health preparedness. Illustrate concepts, methods, and tools of public health data collection, analysis and interpretation, and the evidence-based reasoning and informatics approaches that are essential to public health practice. Demonstrate principles of effective functioning within and across organizations and as members of interdisciplinary and inter-professional teams in addition to respectful engagement with people of different cultures and socioeconomic strata. Apply concepts and principles of program planning, development, budgeting, management, and evaluation in organizational and community initiatives. Apply effective written and oral skills for communicating with different audiences in the context of professional public health activities.

## Discussion

The landscape of public health is changing. While the past decade alone has seen major progress in health, it has also faced the emergence of new health conditions and the reemergence of those once under control. Public health has witnessed genetics and public health biology rise to the forefront of public health practice. Yet, in the wake of these rapid changes, many public health programs are left with dated programs and curricula that no longer reflect the most pertinent needs of the communities we serve and the organizations for which we work.

In this rapidly changing environment, public health professionals, who have been called upon to find ways to better address emerging public health issues and serve as drivers of change, are at a distinct advantage. Public health professionals are trained to be change agents; they trained to assess problems, determine capacity, and develop evidence-informed programs. Simply, taking a public health approach to addressing curricular needs can lead to a stronger, more well-informed public health workforce. In doing so, sustainable, impactful change is almost guaranteed. However, to do so requires public health schools and programs to reflect on current capacity, strengths, and weaknesses. Such a major reform—in thinking, theory, and practice—requires an organized, multistep approach. Intervention Mapping provides an ideal framework for this, allowing a group to access capacity, specify goals, and ultimately guide the entire change process from curriculum development to implementation and evaluation of competency-driven programs.

This multiphase process requires input from critical users—the students, faculty, and employers who will ultimately be affected by this change. As with any transformational process, this process requires considerable time, allowing for input from all stakeholders at each step. Input helps to ensure both a well thought out change process while also helping to ensure buy-in. As the process of Intervention Mapping continues, critical input at each step of the process also provides key stakeholders with the time to observe what works, recognize mistakes, and make adjustments accordingly for a stronger design.

This transformational process is not without challenges, and perhaps the greatest challenges occur in the first steps. It is during this time that the organization must agree to initiate the change process, assess capacity, take a deep look at strengths and weaknesses, and take those first steps toward specifying curricular goals. As with anything, the first steps are the hardest and the slowest. In our example, we were able to quickly assess capacity and current needs. Student demographic trends and evaluation data suggested younger populations of students required more innovative and technologically savvy curricula, while also revealing a desire to have more in-class and fewer online courses. Alumni and employers stressed the need for certain skills that were not foci in the traditional core, including writing, systems thinking, professionalism, and communication. Course success rates and CPH exam pass rates highlighted the need for refocusing curricular endeavors, and meetings with students and faculty alike suggested a desire for fresher content and movement away from a siloed approach to education. This practice was supported by changing ASPPH guidance and 2016 revisions to the CEPH core competencies, which themselves necessitate a complete overhaul to the traditional curriculum.

Still, in light of these supports toward change, numerous barriers existed. The greatest barrier included embarking on a change process while the CEPH competencies were still under revision. The TMPH committee was charged with making changes in light of the impending integrated model but without final competencies to build around. Therefore, the team was trying to meet current criteria, while anticipating future changes. In addition, university system policies regarding credit hours, course scheduling, and graduation requirements favor the traditional, and faculty, themselves, are not generally eager to change. At the onset, there were individuals who thought this daunting task could not be achieved; the barriers to change were too great. Thus, the first steps were the slowest to initiate. The TMPH committee was tasked with rethinking how public health has traditionally been taught and to redesign the landscape of future public health education. Such large-scale transformation of traditional views and ideologies could not be imposed nor could the timeline be pushed. This required iterative conversation and slow progression toward change.

Following faculty readiness and mobilization came the daunting task of developing specific curricular goals. Faculty had to fully examine all alternatives and come to agreement on the guiding principles and processes for the transformation of the MPH core. Again, using research-informed methods (e.g., pile sorting), a common technique used in the qualitative analysis of public health data; modified Delphi techniques (structured communication techniques often used in public health and health policy decision making) helped to streamline this process. Furthermore, it was through the successful completion of these processes, which illustrated redundancies and gaps in the current curriculum that faculty buy-in became widespread. This step also further supported the need for careful and thoughtful approaches toward change to enhance buy-in and participation.

## Conclusion

Ultimately, the steps outlined in this paper resulted in a final set of revised MPH core competencies that are interdisciplinary in nature and fulfill the challenges set forth by the 2002 IOM report (3), the 2014 ASPPH Framing the Future report (7) and changing trends in both public health education and challenges in keeping the public healthy. These competencies were agreed upon by the entire COPH faculty, which signaled the College’s readiness for change and provided the impetus to revolutionize the delivery of public health education at the University of South Florida.

## Author Contributions

All of the authors of this paper contributed to the development and design of the reported curricula, as well as in the development, writing, and editing of this manuscript.

## Conflict of Interest Statement

The authors declare that the research was conducted in the absence of any commercial or financial relationships that could be construed as a potential conflict of interest.
